# Occupational exposure to metals and other elements in the tractor production

**DOI:** 10.1371/journal.pone.0208932

**Published:** 2018-12-14

**Authors:** Denis Vinnikov, Sergey Semizhon, Tatsyana Rybina, Viktor Zaitsev, Anna Pleshkova, Aliaksandra Rybina

**Affiliations:** 1 al-Farabi Kazakh National University, Almaty, Kazakhstan; 2 Biological institute, National Research Tomsk State University, Tomsk, Russian Federation; 3 Minsk Tractor Plant, Minsk, Belarus; 4 National Center of Occupational Safety and Health, Minsk, Belarus; 5 Republican Scientific and Practical Centre of Hygiene, Minsk, Belarus; Chinese Academy of Sciences, CHINA

## Abstract

**Background:**

Exposure to metals via air sampling in workplace has been extensively studied; however, the magnitude of individual exposure in various occupational groups may vary dramatically. The aim of this cross-sectional study was to ascertain exposure to selected metals from metal fumes in a series of typical workplaces of contemporary tractor production.

**Methods:**

Ninety-eight (median age 41 (interquartile range (IQR) 23) years, all men) workers from Minsk Tractor Plant were categorized into four groups, including assembly shop workers (group 1); thermal shop staff (group 2); steelmakers (group 3) and welders (group 4). Hair samples (0.25 g) of each worker were tested for Ca, Mg, P, Cu, Fe, Zn, Al, Mn, Cr, Ni, Pb and Cd using atomic emission spectrophotometry. We then tested between-group differences of log-transformed element concentrations using analysis of variance, followed by logistic regression to determine the odds ratio (OR) with its 95% confidence interval (CI) of high exposure for four selected groups.

**Results:**

The median work duration in workers was 6 (IQR 15) years, more in group 1 (10 (IQR 23)). Eight out of 12 included elements yielded significant between-group differences, including Mg, P, Fe, Zn, Mn, Cr, Ni and Cd. Steelmakers had higher Mn hair concentrations (F-ratio 10.41, p<0.001); whereas Fe (F-ratio 12.48, p<0.001), P (F-ratio 12.68, p<0.001), Zn (F-ratio 6.07, p<0.001) and Cr (F-ratio 20.54, p<0.001) were higher in welders. OR of high exposure to Mg in group 3 was 10.00 (95% CI 1.14–87.52), whereas the OR of high exposure to P in group 4 was 18.64 (95% CI 2.22–156.85) compared to group 1.

**Conclusions:**

In the modern full-cycle tractor production, welders may have higher exposure to Fe, P, Zn and Cr, as opposed to steelmakers with higher Mn hair concentrations.

## Introduction

Occupational exposure to metal fumes and aerosol has been extensively studied [1], especially in welders [2,3]. Studies from different settings report wide ranges of exposure not only when discriminating between welders and other occupations, but even within welders’ strata, to take manganese as an example [4]. Such wide range of exposure may explain heterogeneity in the associated clinical outcomes in those working in the industry, including chronic obstructive pulmonary disease, neuropathy and other occupational diseases. High smoking prevalence, dietary habits and lifestyle may confound such associations, therefore more evidence on the individual exposure are needed to assess risk.

Exposure data, including those from large national-wide studies, rely on various concentrations in the fume or aerosol, but may not reflect personal exposure as a function of absorption and metabolism. Studies may rely on self-reported occupational history [5] and may combine the data with blood metal samples [3], or hair concentration [6] but may provide inconsistent conclusions on the associations when compared to each other [5–7]. Work conditions and settings may differ to a great extent, which hampers exposure data comparison and derivation of occupational exposure limits for selected occupations in the metal industry, such as welders [8].

To further explore the exposure levels of those working in the machine production industry, we have compiled a cohort of workers from one factory having quite similar socioeconomic status and smoking history. The venue for this study, Minsk Tractor Plant (MTP) has a full cycle of production from steelmaking to assembly, therefore, enables to mine exposure data from both low- and highly exposed workers. The aim of this cross-sectional study was to ascertain exposure to selected metals from metal fumes in a series of typical workplaces of contemporary tractor production and to compare metal air concentrations with individual exposure measured as metal concentrations in workers’ hair.

## Materials and methods

### Study venue

This study was approved by the local committees in bioethics from the National Center of Occupational Safety and Health, Minsk, Belarus and al-Farabi Kazakh National University, Almaty, Kazakhstan. Informed consent was obtained from each subject.

MTP is a whole-cycle tractor production facility, producing and selling 40–50 thousand tractors a year and located in downtown Minsk, Belarus. Tractor manufacturing embraces all stages of production, starting with steelmaking shops producing steel, design facilities, assembly, dying, final product testing, as well as all sorts of ancillary departments, such as finance, human resources and even social infrastructure, including kindergarten, own medical out-patient facility for pre-employment and periodic screening and surveillance, and recreational venues. The current study has a cross-sectional design, and initially 98 workers were randomly selected and included in this analysis. The list of workers for randomization was provided by the human resources department, and of the entire sample of 398 workers employed for high-exposure workplaces assessed in this study, we selected every fourth worker from the list (sample 25%). The study sample did not differ from a pilot sample of the remaining and not included workers in terms of age, sex, smoking and work duration. These workers provided their full history, including demographic characteristics and smoking status. Employment history was obtained from the electronic records of human resources department with regard to current position and work duration. Invited workers provided their blood for biochemical analysis (cholesterol, glucose, total protein, transaminases) and hair samples. Personal protective equipment (PPE) use is mandated by the local legislation at all times; however, the supervision and enforcement remain the supervisor’s responsibility. In general, data from safety department support high level of compliance with these regulations, and for the current analysis, we assume that most workers were wearing PPE for the entire workday. PPE in this plant includes worker’s uniform protecting worker’s skin, except face and hands, and a respirator. The worker’s uniform also includes safety boots and plastic protective glasses.

Based on the regular industrial hygiene department analysis and the data supplied to the research team, we grouped workers into four categories of exposure to metal aerosol from almost all production-associated workplaces. Metalworkers in the assembly shop were all included in the first group of the lowest exposure level (group 1); thermal shop operators including metal cleaners were included in the thermal shop group (group 2); steelmakers from the steelmaking shop were included in group 3; all welders from various locations throughout the plant were included in group 4. The choice of elements was guided by a range of elements sampled by the governmental bodies as part of industrial hygiene program and being the main constituents of stainless steel used in production, the type of which remains constant.

### Exposure to metal aerosol assessment

Air sampling was not planned or implemented by the current analysis; therefore, we used industrial hygiene data from the plant to stratify workers into four groups. In brief, industrial air sampling methodology in workplaces is mandated and standardized by the Statement of the Ministry of Health of the Republic of Belarus #92 dated October 11, 2017. This Statement is an update of the earlier version, which we used to collect air samples and analyze metals in the collected dust samples from the air. Air sampling was performed in the breathing zone of each included worker at an elevation of at least 1.5 m from the floor when standing or 1.0 m when sitting. During the workday, four air samples were collected from each worker. We used geometric mean concentrations form the series of measurements to define high- vs. low-concentration workplaces and to guide the stratification of workers into four proposed occupational groups.

### Hair metal concentrations measurements

Hair metal concentrations were elected as markers of exposure in this analysis as opposed to blood concentrations for two reasons. Firstly, hair element concentration measurement is a non-invasive method, easy to use and does not require safety measures associated with blood work. Secondly, in contrast with blood samples, hair concentrations will reflect cumulative, equalized exposure, not limited to a specific day and, thus, minimizes the bias from a single test, since the hair level reflects exposure only limited to the individual period of hair cut. We collected scalp hair from each worker in the morning the day a worker was asked to wash his hair. For comparison, we used certified standard concentration samples. Hair was cut in the occipital area as close to skin as possible with the total mass of at least 0.2 g. Hair samples were then washed using non-ionized surface active solution, following by non-ionized water, then acetone and then again with non-ionized water. We then dried hair at 60 degrees C for 3 hours, and then used microwave mineralizer Mars 5 (CEM, USA) to prepare samples at 1600 W controlling for pressure. Pressure was gradually increased during 20 minutes up to 1.0 MPa and kept at this level for 10 more minutes. Mineralized samples were then put in 25 ml tubes and filled to a mark with distilled water. Ten ml of a 4:1 ratio mixture of nitric acid (67%) with hydrogen peroxide (30%) was used as oxidizer. Metal concentrations in the workers’ samples and reference samples were tested using atomic emission spectrophotometry (Ultima 2, Horiba Yobin Ivon, Japan). We programmed the procedure for simultaneous testing of all elements in one sample, and metal concentrations were measured in mg/kg (equal to mcg/g) of hair. Using the method, the lower limit of detection (LOD) were 0.6; 0.2; 0.45; 3.0; 0.15; 0.2; 0.09 and 0.06 mg/kg for Cu, Fe, Zn, Al, Mn, Cr, Ni and Pb respectively.

### Statistical analysis

Our primary outcome was hair concentration expressed as mg/kg. All data were tested for normality using Shapiro-Wilk test modified in NCSS to allow unlimited sample sizes. The means are shown as either mean ± standard deviation in case of normal distribution or median with the corresponding interquartile range (IQR) otherwise. Most hair concentrations were left-skewed; therefore, we log-transformed hair concentrations for further between-group comparisons. Between-group variance of the cumulative metal log-transformed concentration in hair was tested against within-group variability using analysis of variance (ANOVA) with the subsequent Tukey-Kramer test for multiple comparisons to detect which specific group significantly differed from others. P-value for F-value below 0.05 was treated as a significantly greater variability between groups compared to within the groups. Additionally, we tested the odds of being exposed to high concentrations using logistic regression. For that, we stratified each worker into either a low- or high-concentration group for each element using their 75^th^ percentile as a cut-off value. In the univariate analysis we tested variables, such as working group, age and work duration and then included significantly different predictors as confounders in the logistic regression model, both crude and adjusted. In these analyses, we calculated odds ratios (OR) with their corresponding 95% confidence intervals (95% CI) of high exposure. All tests were run in NCSS 12 (Utah, USA).

## Results

Ninety-eight workers were ultimately included in the analysis. All blood and hair concentrations were left-skewed and non-normally distributed. All subjects in all groups were men, generally older and with longer work duration in the first group (metalworkers of the assembly shop) (Table 1). Smoking status of workers did not differ between groups.

**Table 1:** Descriptive summary data of four included groups of workers.

	All	Group 1 (metalworkers of assembly shop)	Group 2 (thermal shop operators)	Group 3 (steelmakers)	Group 4 (welders)
N (%)	98 (100)	17 (17)	17 (17)	26 (26)	38 (38)
Males, N (%)	98 (100)	17 (100)	17 (100)	26 (100)	38 (100)
Age, years (IQR or SD)	41 (23)	48.1±13.8	36.9±10.4*	37 (19)*	43 (26)*
Work duration, years (IQR)	6 (15)	10 (23)	5 (10)	6 (14)	4 (16)*
Smokers, N (%)	41 (42)	7 (41)	8 (47)	10 (38)	16 (42)
Cigarettes smoked a day^-1^ (SD)	18.1±7.8	16.2±8.1	19.4±6.0	19.0±9.0	17.8±8.8

Note: all concentrations are presented as either means±standard deviations (SD) or medians (interquartile range (IQR)) depending on the distribution:

*—significant difference when compared to group 1.

### Hair concentrations

**Fig 1:**
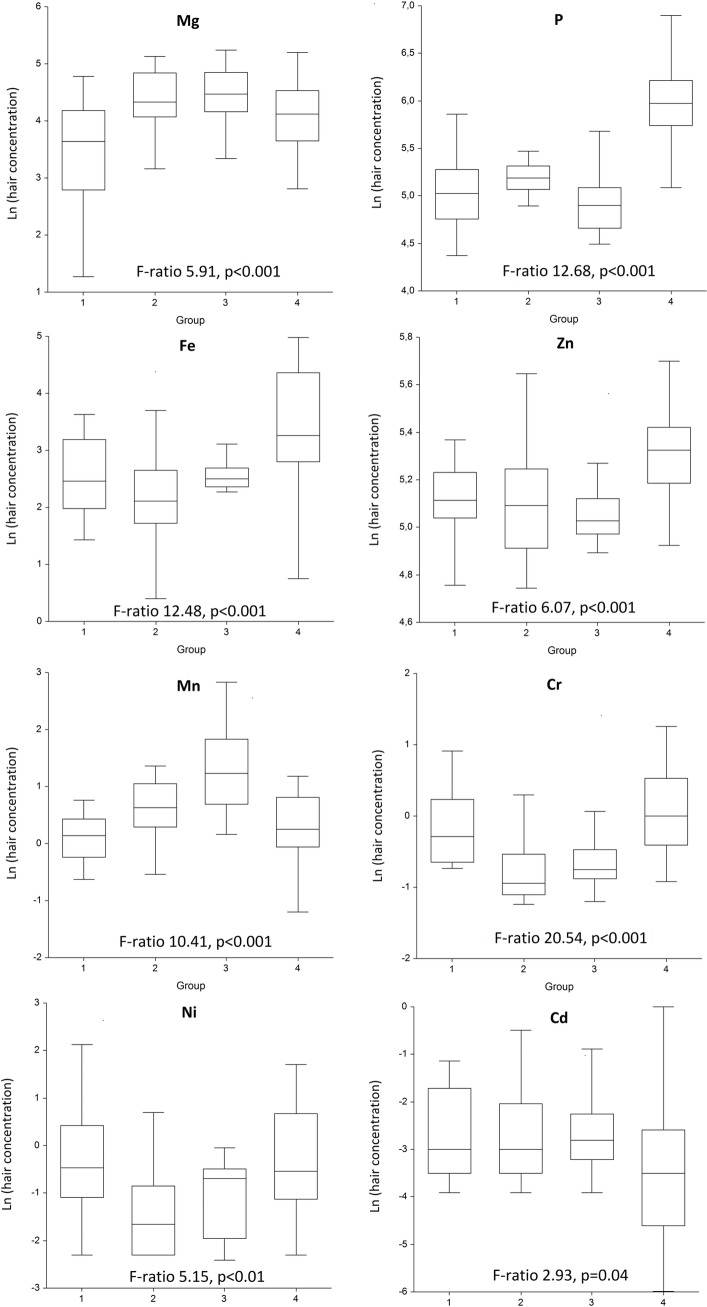
Log-transformed hair concentrations in the groups tested with ANOVA (only elements with p<0.05 shown). Group 1 –metalworkers of assembly shop operators; Group 2 –thermal shop operators; Group 3 –steelmakers; Group 4 –welders.

Twelve elements were analyzed in the hair of workers of selected four workplaces and occupations (Table 2). Hair element concentrations ranged dramatically from 0.05 (IQR 0.08) mg/kg of cadmium to 995 (IQR 915) mg/kg of calcium. Between-group variance did not exceed that of within-group with regard to four elements, including Ca, Cu, Al, and Pb, reflecting non-different personal exposure to these elements in the workers of selected four groups. Hair concentrations of eight elements were significantly different in multiple comparisons. Assembly shop workers’ hair had higher concentration of Cr and Ni; steelmakers had elevated Mn concentrations, whereas welders showed significantly greater concentrations of Fe, P, Zn and Cr in their hair compared to other groups (Tukey-Kramer *post-hoc* test for multiple comparisons, data not shown). The highest F-ratio (F-ratio 20.54, p<0.001) indicating significant difference in Cr concentration when compared to other groups was found in welders (Fig 1). Between-group variances were also very high for P (F-ratio 12.68, p<0.001), Fe (F-ratio 12.48, p<0.001) and Mn (F-ratio 10.41, p<0.001), of which Fe and P concentrations were very high in welders, while Mn was elevated in steelmakers. Moderate, but still significant between-group variances were found for Zn (F-ratio 6.07, p<0.001, greater concentration in welders), Mg (F-ratio 5.91, p<0.001, smaller concentration in assembly shop workers) and Ni (F-ratio 5.15, p<0.01, smaller concentration in assembly shop workers). We could not find direct correlations of industrial hygiene typical workplace air concentrations with personal hair concentrations for most studied elements. Only Fe (r = 0.21; p = 0.04) and Cr (r = 0.40; p = 0.001) showed increased hair concentrations with elevated air concentrations in such analyses.

**Table 2:** Concentrations of metals in the hair of workers from four groups.

	All	Group 1 (metalworkers of assembly shop)	Group 2 (thermal shop operators)	Group 3 (steelmakers)	Group 4 (welders)
Ca, mg/kg (IQR or SD)	995 (915)	979±613	1407±852	1030 (862)	967 (1096)
Mg, mg/kg (IQR or SD)	66.8 (66.2)	44.8±32.0	89.6±44.5	92.8±44.3	61.8 (52.7)
P, mg/kg (IQR)	182 (241)	152 (79)	179 (45)	134 (56)	393 (190)
Cu, mg/kg (IQR or SD)	10.6 (3.4)	10 (3)	10 (2)	11.6±2.5	11 (5)
Fe, mg/kg (IQR)	14.3 (18)	12 (17)	8 (9)	12 (4)	26 (62)
Zn, mg/kg (IQR)	173.0 (58)	166 (33)	163 (54)	152 (23)	205 (48)
Al, mg/kg (IQR)	7 (5)	6 (4)	5 (13)	6.6 (3.4)	8.2 (72)
Mn, mg/kg (IQR)	1.7 (1.8)	1.2 (0.7)	1.9 (1.5)	3.4 (4.2)	1.3 (1.3)
Cr, mg/kg (IQR)	0.6 (0.6)	0.8 (0.8)	0.4 (0.3)	0.5 (0.2)	1 (1)
Ni, mg/kg (IQR)	0.5 (0.6)	0.6 (1.5)	0.2 (0.3)	0.5 (0.5)	0.6 (1.6)
Pb, mg/kg (IQR)	1.0 (1.1)	1.0 (1.0)	0.9 (1.2)	0.9 (1.2)	1.0 (1.3)
Cd, mg/kg (IQR)	0.05 (0.08)	0.05 (0.15)	0.05 (0.1)	0.06 (0.07)	0.03 (0.07)

Eight elements with significant between-group differences were then tested in regression analysis to define the odds of high exposure in selected groups compared to the reference group. When identifying potential confounders, we found that work duration was significantly different between low- and high-exposure groups with regard to P, Fe and Ni, whereas age may confound Cd hair levels. The ORs from such analyses were non-significant for Fe, Zn, Mn, Cr, Ni and Cd, mostly because the groups were too small to attain higher power. Nevertheless, the OR of high exposure to Mg in group 3 was 10.00 (95% CI 1.14–87.52), whereas the OR of high exposure to P in group 4 was 18.64 (95% CI 2.22–156.85) compared to the reference group 1.

## Discussion

This was an occupational exposure study at Minsk Tractor Plant, in which workers with documented exposure history were categorized into four groups of exposure to selected elements. In this analysis, we compared workers from the tractor assembly shop with the presumed least exposure to metals treating them as reference group, with thermal shop, steelmakers and welders within one plant. We found significantly greater concentrations of Fe, P, Zn and Cr in the hair of welders, as opposed to higher Mn concentrations in steelmakers. Because included workers had quite comparable eating habits, i.e. work at one plant and have lunch together in a similar catering site, and smoking status, these elemental hair concentrations likely reflect the differences in the occupational exposure to the main elements between the selected groups.

Measuring metal ion concentration in hair both for environmental and occupational research is a non-invasive methodology of assessing personal exposure. Despite existing methodology and the use for more than 30 years in occupational medicine [9], measuring elemental concentrations in workers’ hair is still not widely used in practice or research, and only a few publications are available to compare elemental hair concentrations in a range of occupations. Hair is easy to obtain, and slow growth rate enables to reflect on more long-term exposure compared to blood, however the method works better for quantification of exposure of groups rather than individuals, and this generally applies to metals as well [10]. Blood and urine concentrations are also widely used in the occupational settings, but the associations of elements’ concentrations in blood and urine with air are in general only observed at higher exposure levels [11]. Hair concentrations may alter as a result of hair washing frequency, smoking, dying, and may also reflect personal eating habits and nutrition profile [5,6], and given whether hair metal concentrations better reflect occupational air sampling is still disputable, metal hair concentrations should be interpreted along with air sampling data to assess occupational exposure to metals. Our findings of somewhat poor reflection of air metal concentrations, reported from the regular industrial hygiene monitoring, in hair confirm the need to combined interpretation of exposure data both from air and hair.

In general, hair concentrations in welders and steelmakers in the current study were lower than in other studies of metal workers, to take Mn in welders as an example (5.78±1.60 mcg/g in [12] vs. median 1.29 (IQR 1.31) mg/kg in the current report). In a study with welding students (welding time 47 min a day), Mn hair concentration ranged from 5.2±8.6 to 1.7±2.1 μg/g depending on the washing method [13]. Although the range is quite close to the concentrations we have in our setting, hair concentrations should be compared only when a standard methodology of measurement is used. In an earlier study in welders [14], a wide range of elements were measured, including some of the elements in the current analysis, and Mn hair concentrations were higher compared to MTP employees (median 5.37 vs 1.29 (IQR 1.31) mg/kg, as opposed to Zn with quite similar hair concentrations (median 188 vs. 205 (IQR 48) mg/kg). Of note, our Mn hair concentrations (median 3.42 (IQR 4.23) mg/kg) in steelmakers were quite close to the ones reported by Luse. Moreover, Cr, Ni and Pb hair concentrations in our steelmakers were lower compared to workers in [15] (5.66±2.21; 3.43±1.16 and 16.21±7.99 mg/g vs. median 0.47 (IQR 0.21); 0.5 (IQR 0.5);and 0.90 (IQR 1.19 mg/kg). For sure, the concentrations may dramatically differ depending on the exposure pattern, setting itself and even hair washing habits, but such lower concentrations may be indicative of lower exposure to selected metals at MTP.

All tested metals in the breathing zone aerosol have specific clinical and well-documented effects. Nickel is a known carcinogen, associated with increased risk of lung cancer, especially water-soluble nickel [16], however the findings are generally inconsistent [17], and the risk may only be elevated in non-smokers [18]. Nickel may also be associated with chronic obstructive pulmonary disease (COPD), as demonstrated in studies from nickel refineries in the Russian North [19]. High concentrations of nickel and nickel tetracarbonyl, way beyond occupational exposure limits (OEL), may be found in nickel ore mining and nickel refineries [19,20], and high exposure to Ni in these studies was associated with COPD [21]. In the breathing zone of workers within those refineries, the mean metallic Ni concentration was 0.15 mg/m^3^ (OEL 0.05 mg/m^3^), which is somewhat comparable to the concentrations regularly reported in the welders’ breathing zone of MTP. Therefore, even in those doing welding at tractor production, the risk of COPD may be elevated and associated with high Ni concentrations, but further research is needed with clear outcome classification and greater sample size. With regard to Cd, we observed almost uniform Cd hair concentrations in four groups of MTP workers, reflecting almost similar air concentrations of this metal throughout the plant. We also believe that such similar hair concentrations may confirm non-different smoking patterns of workers in our groups, as they all were homogenously males with similar smoking status.

Among the studied elements, lead attracted most of researchers’ attention in the past and present, and plenty of studies have documented kidney toxicity [22], cardiovascular effects, including arterial hypertension [23], genotoxicity [24], central nervous system effects [25] and reproductive toxicity [26], such as deterioration in human semen quality even in moderate exposures. The latter study showed similar effect not only for lead, but for Cd as well. Cd exhibits its toxic effects through oxidative stress and its interaction with essential elements zinc and magnesium [27]. Oxidative stress may also be a mechanism of copper toxicity in case of chronic Cu overload [28], which may occur in the occupational settings. Pb and Cd, along with Mn and Zn may have endocrine disrupting properties, but the mechanism is not fully understood [29]. Taken together, chronic exposure to these elements may have a profound effect on the worker’s health, and their monitoring should guide preventive strategies in the occupational medicine.

This study was generally limited to a small sample size. Another limitation was inclusion of only men in this study, which eventually made it impossible to verify exposure levels in women, because sex may affect metal concentrations in hair. In addition, we grouped workers into four categories of exposure, hypothesized *a priori*, and such stratification may have introduced exposure classification bias, but more detailed grouping was impossible because of reduced sample size in each category with more detailed stratification. Because we did not aim to quantify the health effects with the exposure, associated with elements, healthy worker survival effect is unlikely in this report. Finally, we need to note that cross-sectional study design does not allow for continuous monitoring as only a “snapshot” of exposure was available to show the concentrations of selected elements in hair along with industrial hygiene data on air sampling. Such design may not be able to demonstrate the variability in exposure.

This study findings have distinct practical implications. Tractor production and assembly process expose workers to metals in the aerosol they inhale, which was confirmed by high metal concentrations in hair in selected occupations, such as in steelmakers and welders. In assembly shops, metal concentrations in the air may be the lowest, providing relatively safe work conditions for workers. Our study shows that steelmaking and welding occupations expose workers to high concentrations of Fe, P, Zn, Mn and Cr and necessitate strict metal concentrations monitoring in the workplace combined with continuous training and the use of personal protection. One of the approaches could be comprehensive workplace interventions in highly exposed workers, and vitamin supplementation to reduce work absenteeism has been proposed at this plant before as an example, but no analysis was accomplished as to whether such incentives affect productivity yet.

In conclusion, to our best knowledge, this is the first study of hair metal concentrations from a full cycle of modern tractor production. We showed that personal exposure to Fe, P, Zn, and Mn in welders and steelmakers exceed that of assembly staff and thermal shop employees. Modern tractor production may expose workers to lower toxic elements concentrations, but more data from other similar settings are needed to confirm that.

## Supporting information

S1 Table Dataset with primary hair concentrations. (XLSX)
